# Fear of COVID-19, Resilience, and Future Anxiety: Psychometric Properties of the Turkish Version of the Dark Future Scale

**DOI:** 10.3390/jpm13040597

**Published:** 2023-03-29

**Authors:** Murat Yıldırım, Ömer Kaynar, Gökmen Arslan, Francesco Chirico

**Affiliations:** 1 Department of Psychology, Faculty of Science and Letters, Agri Ibrahim Cecen University, 04100 Agri, Turkey; 2 Department of Coaching Education, Faculty of Sports Sciences, Mus Alparslan University, 49250 Mus, Turkey; 3 Department of Guidance and Psychological Counseling, Faculty of EducationMehmet Akif Ersoy University, 15030 Burdur, Turkey; 4 Post-Graduate School of Occupational Health, Università Cattolica del Sacro Cuore, 00168 Rome, Italy; 5 Health Service Department, Italian State Police, Ministry of the Interior, 20127 Milan, Italy

**Keywords:** fear of COVID-19, resilience, future anxiety, Dark Future Scale, Turkish validation

## Abstract

The COVID-19 pandemic has brought many disruptions to individuals’ everyday lives and caused wide-ranging, drastic effects on their well-being, mental health, and physical health. This study sought to validate the Dark Future Scale (DFS) and examine its reliability and validity in Turkish. The present study also examined the relationship between fear of COVID-19, dark future anxiety, and resilience during the COVID-19 pandemic in Turkey. Four hundred and eighty-nine Turkish athletes (mean age = 23.08 ± 6.64) completed measures on fear, anxiety, resilience, and demographic information. Exploratory and confirmatory factor analyses revealed that the DFS had a one-factor solution with good reliability. Fear of COVID-19 significantly predicted resilience and future anxiety. Furthermore, resilience significantly predicted anxiety and mediated the effect of fear of COVID-19 on future anxiety. The findings have important implications for improving mental health and developing the resiliency of athletes during public health crises such as the COVID-19 pandemic.

## 1. Introduction

The coronavirus disease (COVID-19) is one of the devastating issues that people around the globe have faced in the 21st century. It does not only harm the physical health of people but mental health, as well, across the globe. The first case of COVID-19 was reported in China and spread to the entire world. According to the daily report published by the World Health Organization [1], as of 16 March 2023, there have been more than 760,360,950 confirmed cases of COVID-19 that include more than 6,873,475 deaths. A total of 13,233,862,804 vaccinations were conducted across the globe as of 14 March 2023. Since the first confirmed case of COVID-19 in Turkey on 11th March 2020, a variety of swift measures have been implemented to control the spread of the disease. For example, converting regular classes to distance learning, stay-at-home orders, quarantines, and partial lockdowns are just some of the measures that have taken place in Turkey during the COVID-19 pandemic. Despite all preventive measures, as of 16 March 2023, there have been more than 17 million infected cases of COVID-19 and more than 101,400 deaths with a total of 139,694,693 vaccine doses (as of 29 January 2023) being administered in Turkey [1].

The prevention of the psychological impacts of the COVID-19 pandemic on individuals and society has been a primary topic of interest among researchers across the globe. Studies have shown that COVID-19 could trigger a wide range of psychosocial and mental health problems such as depression, anxiety, stress, panic disorders, and fear [2,3,4,5] and, consequently, possess a serious threat to individuals’ lives and physical health. In addition, the aftermath of the pandemic has been linked to mental health in the general population [6,7], healthcare professionals [8], students [9,10,11,12], and athletes [13]. For example, a study conducted in Turkey reported that fear of COVID-19 could increase levels of anxiety, depression, and stress that reduce the level of resilience [14]. Another study found that fear of COVID-19 was associated with increased perceived risk and anxiety and diminished mental well-being [15]. Accordingly, this has triggered a great deal of interest in how individuals may cope with their dysfunctional fear related to COVID-19 and protect their mental health, thereby increasing the importance of psychological resources (e.g., resilience) and coping mechanisms in the face of adversity.

Future anxiety refers to the experience of tension, uncertainty, and anxiety corresponding to potentially unhealthy events to come [16]. The intensity and degree of future anxiety are related to a wide range of factors, including the importance of the predicted event in the future, the anticipated probability of its occurrence, and individuals’ beliefs and capacity to deal with the event. Future anxiety can affect the attitudes, cognitive processes, and behaviors of an individual [17]. A high level of future anxiety is associated with avoidance, uncertainty, passivity, and poor planning for the future [18]. Deterministic beliefs about the present time and dysfunctional beliefs about one’s past are significantly related to future anxiety [16]. Due to fear, uncertainty, worry, and anxiety related to COVID-19, people may suffer from high levels of future anxiety, which may, in turn, affect the overall psychological functioning of individuals and diminish coping strategies to deal with distress in times of health crisis [19]. Studies carried out during the COVID-19 pandemic provided evidence supporting the link between future anxiety and health-related outcomes. For example, Duplaga and Grysztar (2021) examined the relationship between future anxiety, health literacy [20], perceived health threat related to COVID-19, and COVID-19-related conspiracy belief. Their results indicated that those who have high levels of perceived health threat related to the COVID-19 pandemic and COVID-19-related conspiracy beliefs and a low level of health literacy are more likely to experience a high level of future anxiety. Another recent study demonstrated that future anxiety had a fully mediating role in the association between COVID-19 victimization experience and mobile phone addiction. Mindfulness was found to moderate the impact of COVID-19 victimization experience on young adults’ future anxiety [21]. With regard to the positive psychological variables, a higher level of self-efficacy was found to be related to a lower level of future anxiety [22], suggesting enhancing self-efficacy may continue to reduce the levels of future anxiety. Based on these findings, it can be plausible to assume that psychological resources, such as resilience, may facilitate a buffering effect against future anxiety. 

## 2. Resilience as Mediator

Resilience is defined as one’s ability to cope with or adaptively regain from stressors [23]. Resilience is a critical psychological resource in sports because athletes should use various mental qualities to resist sports-related pressures [24]. These mental qualities allow athletes to positively adapt to stressors and prevent psychological harm to provide high-quality performance. Individuals with a low level of resilience may suffer from higher challenges in dealing with adversities or mental health problems [25]. Protective factors serve a vital role in preventing mental health challenges, and psychological factors, such as resilience, are one of the primary protective factors in psychological health [26]. In addition, resilience protects individuals against adverse psychological outcomes [23] and has been found to be a central protective and mitigating factor for functional responses in times of stressful situations, such as the current COVID-19 pandemic [27]. Furthermore, resilience has been examined as a mediator. For example, it has been suggested that resilience mediates the associations between fear of COVID-19 and trait resilience [10] and between COVID-19 stress and COVID-19 burnout [28] alongside promoting better mental health [29]. Therefore, it is tenable to assume that resilience can mediate the association between fear of COVID-19 and future anxiety among athletes. 

Given the above-mentioned theoretical and empirical evidence, the current study endeavors to extend the earlier literature by examining two primary objectives. Firstly, we investigate the psychometric properties of the Dark Future Scale (DFS) in Turkish to enhance the utility of the scale both for research and practice. Secondly, this study examines the mediating role of resilience in the relationship between fear of COVID-19 and future anxiety among Turkish athletes during the public health crisis of the pandemic. Understanding the underlying mechanism between fear and future anxiety would be useful to tailor and implement effective interventions targeted at increasing resilience and decreasing the mental health problems of athletes. Our study process is structured around the following hypotheses: (i) the DFS will show adequate psychometric properties in Turkish; (ii) fear of COVID-19 will have a direct effect on resilience and future anxiety; (iii) resilience will have a direct effect on future anxiety; and (iv) resilience will mediate the relationship between fear of COVID-19 and future anxiety. 

## 3. Method

### 3.1. Measures

*Fear of COVID-19 Scale (FCV-19S)*: The FCV-19S was developed to assess dysfunctional fear related to the COVID-19 pandemic [2]. The scale includes seven items rated on a 5-point Likert-type scale ranging from 1 (strongly disagree) to 5 (strongly agree). A sample item is “I am afraid of losing my life because of the coronavirus”. A high score on the FCV-19S indicates a greater experience of fear of COVID-19. The FCV-19S demonstrated good reliability and validity evidence in Turkish [30]. The internal reliability estimate with the current study was 0.87. 

*Dark Future Scale (DFS):* The DFS was used to measure concern and anxiety toward the future [16]. The DFS is a 5-item scale (e.g., “I am afraid that the problems which trouble me now will continue for a long time”) with scoring based on a 7-point Likert-type scale, varying from 0 (decidedly false) to 6 (decidedly true). A high score on the DFS reflects greater levels of future anxiety. The DFS yielded an acceptable internal reliability estimate in the current study (α = 0.79). Further psychometric properties of this scale were examined in this study.

*Brief Resilience Scale (BRS):* The BRS was developed to measure a person’s ability to deal with challenging situations and recover from stress and adversity [31]. The BRS includes six items answered on a 5-point Likert-type scale ranging from 1 (strongly disagree) to 5 (strongly agree). A sample is “It is hard for me to snap back when something bad happens”. A high score on the BRS signifies a greater ability to overcome difficulties. The Turkish version of the BRS showed satisfactory psychometric properties for the scale [32]. The BRS had an acceptable internal reliability estimate in the current study (α = 0.70).

### 3.2. Study Procedure

This study used a convenience sampling method to collect data. A web-based survey was created including the demographic information and measures of the study. Prior to taking part in the online survey, informed consent was obtained from all participants. They were ensured of the anonymity and confidentiality of responses as well as being fully informed about their rights to participation. A forward–backward approach to translation was utilized to ensure that DFS made sense in the Turkish language. To that end, five independent bilingual academics contributed to the final form of DFS in Turkish culture, with three of them involved in forward translation and two of them involved in back translation.

### 3.3. Data Analysis

To examine the factor structure of DFS, the entire sample (*n* = 489) was split into two subsamples. The first subsample (*n* = 244) was used to perform exploratory factor analysis (EFA), and the other subsample (*n* = 245) was utilized to conduct confirmatory factor analysis (CFA). The maximum likelihood method was used both in EFA and CFA. For the main analysis, a two-step analytic approach was followed. In the first step of the analysis, descriptive statistics, reliability estimates, and correlation coefficients were computed. Normality assumption was also explored using skewness and kurtosis scores with their values < |1| = good for normality [33]. In the second step of the analysis, the PROCESS macro (Model 4) for SPSS version 3.5 [34] was performed to test the mediating effect of resilience on the association of fear of COVID-19 and future anxiety. The results of mediation analysis were evaluated using standardized regression estimates (β) and squared-multiple correlations (*R*^2^) with conventional effect sizes: 0.01–0.059 referring to small, 0.06–0.139 referring to moderate, and 0.14 referring to large [35]. A bootstrap approach with 5000 resamples to estimate the 95% confidence intervals was also performed for assessing the indirect effect. The bootstrapping method of 5000 resamples was used to estimate the indirect effect for calculating a 95% confidence interval. All data analyses were carried out using SPSS version 24.0 and AMOS version 24.0 for Windows.

## 4. Results

### 4.1. Characteristics of Participants

Participants (*n*  =  489) were young athletes living in Turkey. They ranged in age between 18 and 68 years *(M*  =  23.08, *SD*  =  6.64). Participants were predominantly males (62.17%), single (87.93%), university graduates (74.64%), not infected with COVID-19 (81.39%), and unvaccinated (75.26%). Most of them were non-national (87.93%) and had been involved in sports between 1 and 3 years (26.79%), with 30.06% of them having the highest achievement of regional champion. A detailed description of the participants’ characteristics is given in Table 1.

### 4.2. Factor Analysis

Using maximum likelihood, we first conducted the EFA to identify the underlying factor structure of DFS. The results show that the Kaiser–Meyer–Olkin value was 0.81, and Bartlett’s test of sphericity value was 332.50 (*p* < 0.001), supporting the sufficiency of data for factor analysis. The analysis yielded a one-factor solution with an eigenvalue of 2.75, accounting for 54.99% of the total variance. Factor loadings ranged between 0.55 (item 2) and 0.73 (item 5).

We then used the CFA to test the measurement model. The latent future anxiety variable was described by its five indicators. We utilized multiple statistics to evaluate the model fit: the standardized root mean square residual (SRMR), the root mean square error of approximation (RMSEA), the relative chi-square (CMIN/DF), the Tucker–Lewis index (TLI), and the comparative fit index (CFI), as well as the chi-square and degrees of freedom. Studies have proposed that values <0.6 and <0.8 for the SRMR and RMSEA, >0.90 for the TLI and CFI, and <3 for CIMIN/DF refer to good model fit, respectively [36]. The results of the measurement model initially produced a poor fit: χ^2^ (5) = 33.298, *p* < 0.001, CIMIN/DF = 6.660, CFI = 0.914, TLI = 0.828, RMSEA = 0.152, and SRMR = 0.058. To improve the model fit, we covaried items 4 and 5 based on the modification indices. The results yielded a good model fit: χ^2^ (4) = 8.778, *p* > 0.05, CIMIN/DF = 2.194, CFI = 0.985, TLI = 0.964, RMSEA = 0.070, and SRMR = 0.028. The standardized factor loadings ranged from 0.56 (item 4) to 0.76 (item 3).

### 4.3. Descriptive Statistics and Correlation Analysis

Prior to testing the mediation model, descriptive statistics, reliability, and correlation coefficients were estimated. Descriptive statistics for each variable showed that skewness and kurtosis values ranged between −0.49 and 0.54, indicating a relatively normal distribution. Internal consistency reliability estimates for the DFS were α = 0.79 both for subsamples and the overall sample. Satisfactory reliability estimates were also found in the other two scales used in this study (see Table 2). Correlation analysis revealed that fear of COVID-19 was negatively correlated with resilience and positively correlated with future anxiety. Further, there was a significant and negative correlation between resilience and future anxiety (see Table 2).

### 4.4. Model Testing

**Figure 1:**
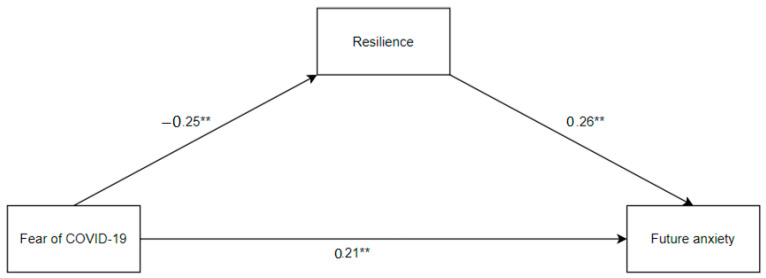
Mediation model depicting the associations between the variables. ** Correlation is significant at the 0.01 level (2-tailed).

A simple mediation analysis was carried out to investigate the mediating effect of resilience on the relationship of fear of COVID-19 with individuals’ future anxiety. The results of mediation analyses revealed that fear of COVID-19 (β = −0.25, *p* < 0.001) had a significant negative effect on resilience by explaining 6% of the variance in resilience. Fear of COVID-19 (β = 0.21, *p* < 0.001) had a significant positive effect on future anxiety, and resilience (β = −0.26, *p* < 0.001) had a significant negative effect on future anxiety. Fear of COVID-19 and resilience collectively explained 14% of the variance in future anxiety. The indirect predictive effect of fear of COVID-19 on future anxiety through resilience was significant, as the 95% confidence interval did not include zero (β = 0.08, 95% CI [0.04, 0.12]). Standardized and unstandardized predictive impacts demonstrating the relationships between the predicted variables are illustrated in Figure 1 and Table 3, respectively. This means that resilience is an important psychological resource in mitigating the adverse impacts of fear of COVID-19 on future anxiety among Turkish athletes during the global health crisis.

## 5. Discussion

The COVID-19 pandemic is not only leading to threats to the physical health of people, but it is also an important risk factor threatening psychological distress (e.g., anxiety) across the globe. Most available research, in the context of the COVID-19 pandemic, has focused on understanding the psychological health of individuals in the current circumstances by assessing the global level of anxiety symptoms and not future anxiety. The main aim of this study was to test the proposed mediation model to explain the mediating role of resilience in the association between fear of COVID-19 and future anxiety in Turkish athletes. The study also tested the psychometric properties of the DFS in Turkish. The findings are discussed below.

The results regarding the psychometric properties of the newly adopted DFS in Turkish confirm that the DFS is a valid and reliable instrument measuring dysfunctional anxiety related to the future by producing a satisfactory internal consistency reliability estimate. The construct validity of the DFS yielded a unidimensional structure including five items. The scale also demonstrated good evidence of convergent validity by showing a positive correlation between fear of COVID-19 and a negative relationship with resilience. These results are in line with previous findings [16,17,20]. However, the findings of this study regarding the factor structure of the DFS were not in accordance with some of the previous research outcomes (e.g., Jannini et al., 2022 [37]). For example, the Italian validation of the DFS was found to have a two-factor structure (internal and external features of anxiety) that showed satisfactory psychometric properties, as indicated by excellent internal consistency reliability and divergent and convergent validity for both research and clinical settings [37].

The results of the correlation analysis indicated that fear of COVID-19 negatively influenced athletes’ resilience and positively affected future anxiety. When an athlete experiences dysfunctional fear related to COVID-19, he/she feels less resilient in the face of adversity and tends to suffer from unhealthy anxiety toward the future [38]. In support of these findings, earlier research found that positive psychological resources (e.g., hope and life orientation) were related to lower future anxiety [17,39].

Most importantly, the subsequent aim of this research was to investigate the mitigating role of resilience in elucidating the underlying mechanism between fear of COVID-19 and future anxiety during the pandemic. The results typically confirmed the associations between fear of COVID-19, resilience, and future anxiety. Athletes who experience fear related to COVID-19 significantly influence their ability to cope with adversities. Consistent with earlier research, fear of COVID-19 was found to be associated with resilience [40], and resilience was found to be linked with future anxiety [38]. Despite limited evidence regarding the link between the analyzed variables, Paredes et al. (2021) reported a moderating role of resilience in the association between perceived COVID-19 threat and future anxiety. A high level of psychological resilience was found to be significantly associated with a lower level of future anxiety [8] and general anxiety [41]. Another study by Rabei et al. (2020) found that self-efficacy is significantly negatively related to future anxiety [22]. Despite the scarcity of evidence, studies in the context of the COVID-19 pandemic also indicated a high level of future anxiety among young individuals due to COVID-19-related stressors [42].

### 5.1. Implications of the Findings

This study contributes to existing knowledge on the mental health of athletes during the pandemic. The present study has four contributions. First, even though some research has examined the association between fear of COVID-19, future anxiety, and resilience, jointly or separately, we empirically confirmed a proposed mediation model with all three study variables simultaneously. Second, using athletes as the research participants, we contributed to the role of resilience in mitigating future anxiety and its implication in understanding athletes’ mental health, an understanding that has yet to be understudied in the Turkish context. Third, most previous research has focused on the mental health of individuals as an independent or dependent variable at a general level. This research supported earlier studies grounding that fear of COVID-19 is the antecedent for resilience and has significant influences on future anxiety. However, due to the cross-sectional nature of this study, it is hard to verify causal links between the variables, which require further validation. Fourth, this research confirmed the theoretical arguments and empirically indicated the associations between fear of COVID-19, resilience, and future anxiety, which satisfied the urgent call in the mental health literature. Fifth, we have shown the crucial role of resilience as a partial mediation between fear of COVID-19 and future anxiety. This highlights the importance of resilience among athletes in reducing future anxiety. This would ultimately help in the development of strategies for athletes’ psychological health.

Our theoretical model has important practical implications for sports coaching. The promotion of athletes’ mental health is of prime concern in sports coaching, particularly in certain situations or activities. Our results yielded the critical role of resilience in fostering positive mental health by reducing the impact of dysfunctional fear on future anxiety in the face of adversity, which would provide crucial inputs for tailoring effective intervention strategies for athletes’ mental health in the era of COVID-19. Therefore, sports coaching must focus on the predictors of athletes’ resilience and its outcome in order to improve their psychological health. Sports coaching must provide appropriate psychological support and fulfill the psychological expectations of athletes, which would empower and encourage them to perform better in their sports with high productivity. In turn, this would positively influence athletes to practice effective strategies, such as resilience, ultimately ensuring better psychological health.

### 5.2. Limitations and Future Directions

The current study has several limitations that need to be taken into account in future research. First, this study used a cross-sectional research design. Although the PROCESS macro examined the association between variables in the proposed model, the findings should be inferred with caution. Subsequent research should be conducted based on longitudinal research design to gain possible support for a causal link between the variables. Second, the sample population was limited to young athletes who were mostly non-national with the highest achievement of regional champions in Turkey. Therefore, the generalization of the findings to athletes performing in different sports may not be possible. As such, subsequent research could replicate the present findings with a diverse sample from different sports. Third, our findings imply that resilience serves an important role in influencing future anxiety. Future research could investigate other possible mitigating psychological resources on anxiety. Other than mediation analysis, a moderator analysis using demographic and psychological variables could be tested in future research.

## 6. Conclusions

In conclusion, Turkish validation of the DFS showed good psychometric properties, indicating adequate internal consistency reliability, good one-factor structure, and convergent validity. Therefore, the Turkish version of the DFS may be viewed as a reliable measurement tool for assessing future anxiety in future research. Additionally, this study tested the mediating role of resilience using fear of COVID-19 as an antecedent and future anxiety as a dependent variable. The results show that the ability to bounce back from adversity would diminish the negative impact of fear of COVID-19 on future anxiety. This would underscore the importance of resilience toward the promotion of mental health in athletes. Therefore, sports coaching could potentially use the results of this study as a guide to promoting athletes’ mental health.

## Figures and Tables

**Table 1:** Sample characteristics (*n* = 489).

Variable	Level	*n*	%
Gender	Male	304	62.17
	Female	185	37.83
Marital status	Single	430	87.93
	Married	56	11.45
	Widowed/separated	3	0.61
Education level	High school and below	98	20.04
	Undergraduate	365	74.64
	Postgraduate	26	5.32
Infected with COVID-19	Yes	91	18.61
	No	398	81.39
Vaccination status	Yes	368	75.26
	No	121	24.74
Active sport year	1–3 years	131	26.79
	4–6 years	126	25.77
	7–9 years	114	23.31
	10–12 years	67	13.70
	13–15 years	23	4.70
	16 years and above	28	5.73
Level of nationality	National	59	12.07
	Non-national	430	87.93
The highest achievement in sport	Regional champion	147	30.06
	National champion	89	18.20
	European champion	8	1.64
	World champion	6	1.23
	Olympic championship	1	0.20
	International tournament	23	4.70
	None	215	43.97

**Table 2:** Descriptive statistics, reliability, and correlations (*n* = 489).

	Descriptive Statistics				Reliability	Correlations		
Variable	Mean	SD	Skew	Kurt	α	1.	2.	3.
1. Fear of COVID-19	15.02	6.03	0.54	−0.22	0.87	1	−0.25 **	0.28 **
2. Resilience	16.79	4.36	−0.18	−0.25	0.70		1	−0.31 **
3. Future anxiety	23.00	7.15	−0.49	−0.43	0.79			1

** Correlation is significant at the 0.01 level (2-tailed).

**Table 3:** Unstandardized coefficients for the mediation model (*n* = 489).

	Consequent			
	*M* (Resilience)			
Antecedent	Coeff.	*SE*	*t*	*p*
*X* (fear of COVID-19)	−0.18	0.03	−5.68	<0.001
Constant	19.49	0.51	37.95	<0.001
	*R*^2^ = 0.06 *F* = 32.22; *p* < 0.001			
	*Y* (Future anxiety)			
*X* (fear of COVID-19)	0.25	0.05	4.85	<0.001
*M* (Resilience)	−0.42	0.07	−5.86	<0.001
Constant	26.26	1.61	16.32	<0.001
	*R*^2^ = 0.14 *F* = 38.41; *p* < 0.001			
Path	Effect	BootSE	BootLLCI	BootULCI
Fear of COVID-19 → resilience → future anxiety	0.08	0.02	0.04	0.12

## Data Availability

The data that support the findings of this study are available from the corresponding author (MY), upon reasonable request.
